# Sporadic, late-onset, and multistage diseases

**DOI:** 10.1093/pnasnexus/pgac095

**Published:** 2022-06-22

**Authors:** Anthony J Webster, Robert Clarke

**Affiliations:** Nuffield Department of Population Health, Big Data Institute, University of Oxford, Old Road Campus, Oxford OX3 7LF, UK; Nuffield Department of Population Health, Big Data Institute, University of Oxford, Old Road Campus, Oxford OX3 7LF, UK

**Keywords:** multistage, aging, risk factors, multimorbidity, epidemiology, somatic mutations, UK Biobank

## Abstract

Multistage disease processes are often characterized by a linear relationship between the log of incidence rates and the log of age. Examples include sequences of somatic mutations, that can cause cancer, and have recently been linked with a range of non-malignant diseases. Using a Weibull distribution to model diseases that occur through an ordered sequence of stages, and another model where stages can occur in any order, we characterized the age-related onset of disease in UK Biobank data. Despite their different underlying assumptions, both models accurately described the incidence of over 450 diseases, demonstrating that multistage disease processes cannot be inferred from this data alone. The parametric models provided unique insights into age-related disease, that conventional studies of relative risks cannot. The rate at which disease risk increases with age was used to distinguish between “sporadic” diseases, with an initially low and slowly increasing risk, and “late-onset” diseases whose negligible risk when young rapidly increases with age. “Relative aging rates” were introduced to quantify how risk factors modify age-related risk, finding the effective age-at-risk of sporadic diseases is strongly modified by common risk factors. Relative aging rates are ideal for risk-stratification, allowing the identification of ages with equivalent-risk in groups with different exposures. Most importantly, our results suggest that a substantial burden of sporadic diseases can be substantially delayed or avoided by early lifestyle interventions.

Significance Statement Modern statistical methods such as logistic regression, characterize the influence of potential risk factors in terms of relative risks. As a consequence, modern epidemiology has tended to ignore the influence of risk factors on aging rates or age-related patterns of disease. By parametrically modeling the incidence of over 450 diseases, we characterized the age-related incidence of disease, and identified over 100 common diseases whose “sporadic” incidence was only weakly age-dependent. Furthermore, we explored how common risk factors modify our effective age-at-risk of these diseases, and our results suggest that early lifestyle changes might substantially delay or even prevent their onset.

Many common diseases are thought to have a multistage aetiology (1–12), in which two or more processes are rate-limiting steps that determine the rate of disease onset. For example, these might include one or more genetic mutations (5,13–18), which was the original reason for using multistage models to describe the onset of cancer (2,3). An important recent discovery is that somatic mutations are prevalent in the majority of tissues in our body (14,19–22). The best known examples include clonal expansions that co-exist with healthy cells in our blood (14,15), skin (19), and esophagus (20). This raises the possibility that somatic or epigenetic mutations could contribute to the initiation of other non-cancerous diseases (13–18,23). This includes generic processes such as *inflamaging* (24,25), and entire classes of diseases, including autoimmune and cardiovascular diseases (13–18, 23). Rates of somatic mutations have also been linked to the lifespan of several different species (26), emphasizing their potential importance for longevity and health. If mutations are involved in triggering disease, then it is likely that they might constitute one or more rate-limiting steps for disease onset, in a similar way to cancer incidence. In that case, multistage models of disease would be expected to describe a much broader range of diseases than just cancer. Here, we found this was true of many common diseases in the UK Biobank cohort (27), and the models provided important insights about the age of onset of these diseases.

We were primarily interested in common age-related diseases that usually occur at ages over 45 y, as would be observed in UK Biobank data. We do not consider rare diseases, that can be strongly influenced by rare germline genetic variants. The mean age at enrolment of participants in the UK Biobank was 57 y (SD, 8 y) (27), and their hospital episode statistics were used to identify the onset of disease. We were interested in disease onset that arises independently of (unconfounded by), other prior diseases, and as in previous work (28), use “incidence” to describe the time to an individual’s first disease in each chapter of the International Classification of Diseases version 10 (29). This is discussed further in the next section and in the Supplementary Material. A total of 800 diseases were studied using two simple parametric models of multistage disease processes, both of which accurately described the incidence rates for approximately 450 of the diseases considered.

In addition to potential biological insights, the parameterized models provided important new insights into disease incidence, that would not be apparent from conventional studies using relative risks. We identified late-onset diseases that appear to become inevitable at the extreme ends of observed human lifespan, and diseases that are more sporadic, often occurring at younger ages, but only weakly influenced by age and potentially avoidable. The incidence of these “sporadic” diseases appears to be particularly sensitive to lifestyle interventions and modifiable risk factors, suggesting that their incidence may be substantially reduced.

One striking observation was that “all diseases are rare,” in the sense that without germline genetic abnormalities the probability *S*(*t*) of surviving for a typical human lifetime (in the survival analysis sense of not getting the disease), is never much less than 1. However, despite the risk of any single disease being relatively low (30), the large number of potentially fatal diseases means that living to old age is unlikely. Consequently, it is often reasonable to approximate the probability density for disease incidence *f*(*t*) by its hazard function *h*(*t*) (28), with *f*(*t*) ≃ *h*(*t*). The Supplementary Material provides data and calculations to justify these approximations, and to support other remarks in the main text.

## Multistage models

**Fig. 1.**
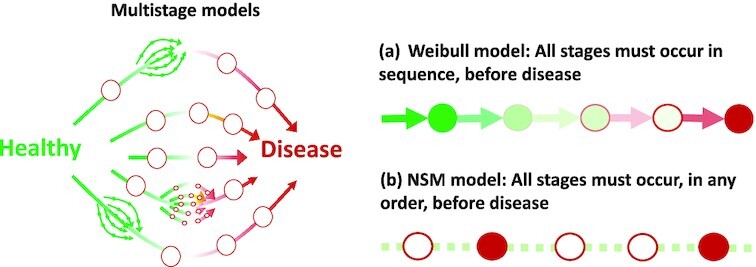
A multistage model can describe any process that arises through one or more independent pathways, with one or more sequential or non-sequential steps. (a) The sequential Armitage–Doll (Weibull) model requires that stages occur sequentially. (b) The non-sequential model (NSM), allows the stages to occur in any order.

The multistage (or “multistep”) model was inspired in the 1950s by a biological model in which cancer involves several genetic mutations before symptoms are observed (1–3, 31). The models describe any process that can arise through one or more independent pathways, with one or more sequential or non-sequential steps (28) (Fig. 1). Whereas proportional hazards models are ideal for studying associations with risk factors, multistage models are ideal for characterizing incidence rates. They provide a concept of relative aging rate, that for a specific disease, captures the relative ages of exposed compared to unexposed individuals. Other advantages include an interpretation in terms of rate-limiting processes, that if biological in origin, might be targeted to slow or prevent disease. They also allow incidence rates to be predicted and extrapolated beyond the observed data, allowing unique insights about disease incidence that cannot be captured by histograms or studies using (non-parametric) proportional hazards methods (Professor Sir David Cox devised the proportional hazards model, and anticipated several points in this paper. In a 1994 interview, he remarked (32), “I would normally want to tackle problems parametrically,” because, “various people have shown that the answers are very insensitive to the parametric formulation of the underlying distribution.”) Throughout, we use the usual notation of a cumulative probability *F*(*t*), survival function *S*(*t*) = 1 − *F*(*t*), probability density function *f* = *dF*/*dt* = −*dS*/*dt*, hazard function *h*(*t*) = *f*(*t*)/*S*(*t*), cumulative hazard function $H(t)=\int _0^t h(s)ds$, and these are related through *S*(*t*) = exp ( − *H*(*t*)) or equivalently *H*(*t*) = −log (*S*(*t*)).

Two models are considered here (Fig. 1a and 1b). The first approximates a sequential multistage model of disease (Fig. 1a), with a Weibull model. As shown in the Supplementary Material, this approximation is good when the probability of having the disease is small over a typical human lifetime, as is the case here. The Weibull model has a proportional hazards form (33), and its hazard function *h*(*t*) can be written as, $h(t)=e^{\eta _X}h_0(t)$, where *t* is time or age, *h*_0_(*t*) = (*t*/*L*)^*m*^ with *m* and *L* (non-integer) estimated parameters, η_*X*_ = ∑_*j*_β_*j*_*X_j_* with *X_j_* risk factors, and β_*j*_ estimated coefficients. Its survival function is

(1) $$\begin{eqnarray*}
\mathit{S}(\mathit{t}) =\exp \left( - e^{\eta _X} \left( \frac{\mathit{t}}{\mathit{L}} \right)^\mathit{m} \right).
\end{eqnarray*}
$$

For traditional multistage models with time-independent rates at each stage, then *m* is an integer (2,28). If the rates at each stage are time-dependent then *m* can be non-integer (28), and *m* is best regarded as an “effective number of steps.” When competing risks (33) exist then *S*(*t*) is the cause-specific survival function, that would differ from the directly observed survival data because e.g. death from a different cause could occur first. These, and several other points mentioned above and throughout the article, are discussed in more detail in the Supplementary Material. The exponent of *S*(*t*) is the cumulative hazard function *H*(*t*) = ∫^*t*^*h*(*s*)*ds*, and can be rearranged as

(2) $$\begin{eqnarray*}
H(t)= \left( \frac{te^{\frac{\eta _X}{m}}}{L} \right)^m,
\end{eqnarray*}
$$

**Fig. 2.**
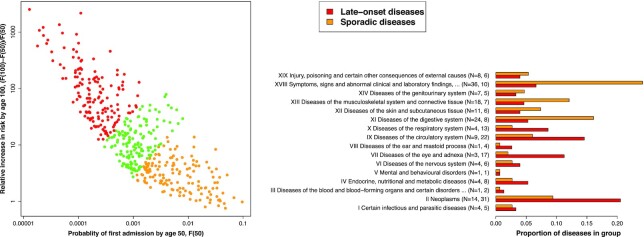
Left: For each disease a dot indicates the probability of first hospital admission at 50, plotted versus the relative increase in probability of disease by age 100. Disease incidence was classified in tertiles to indicate how sporadic or late-onset it was (see main text). Results for the Weibull model are shown, the NSM model gave similar results (Supplementary Material). Right: The composition of sporadic and late-onset diseases in terms of ICD-10 chapters, with N the number of diseases.

This gives an effective age $t e^{\frac{\eta _X}{m}}$, that is determined by risk factors *X*, with the factor $e^{\frac{\eta _X}{m}}$ giving a relative aging rate compared with the baseline values where η_*X*_ = 0 and $e^{\frac{\eta _X}{m}}=1$. It also emphasizes that for any given disease, the influence of risk factors on your aging rate through η_*X*_, will be suppressed by a factor of 1/*m*. For multistage models with an effective number of steps (28) *m*, the relative aging rate will tend to be smaller for larger *m*. Also, when risk factors can be modeled in a proportional hazards framework, they will only modify the time-scale *L* to $Le^{\frac{-\eta _X}{m}}$, leaving *m* unchanged. This means that ratios that only depend on *m*, such as *H*(100)/*H*(50) = (100/50)^*m*^ and the vertical axis of Fig. 2, may be less sensitive to cohorts having systematically different unmeasured risk factors.

The second model considers multistage processes that can occur in any order (Fig. 1b), and takes all events to happen with approximately constant rates. This is a reasonable approximation, because within a non-sequential model all the rate-limiting steps must have similarly slow rates, or they would not be rate-limiting and observable from the incidence data. This mathematical fact is discussed further in the Supplementary Material. We refer to this non-sequential model as the “NSM” model, and its survival function is (see Supplementary Material)

(3) $$\begin{eqnarray*}
S(t)= 1- \left( 1 - e^{-t/L} \right)^m,
\end{eqnarray*}
$$

where *m* is the effective number of steps, *t* is age, and *L* is a time scale for the disease processes. This model has a very different biological interpretation than the Weibull model, typically with a much larger number of steps.

The Weibull and NSM models are derived in the Supplementary Material, along with several relevant observations that have not been published elsewhere. One (important) counter-intuitive observation, is that even when a disease proceeds sequentially through a series of *m* steps, if the rate of one or more steps increase sufficiently, then the observed number of steps will decrease, despite the associated biological processes continuing to occur. Although not necessarily obvious, the mathematical result is very clear. Another observation is that it will often be reasonable to study multistage disease processes with a proportional hazards model. If the hazard of every stage can individually be approximated by a proportional hazards model, then for the “rare” diseases that we study here, a multistage disease can also be described by a proportional hazards model. However, the estimated associations are *β* = ∑_*i*_*β_i_*, a sum over the associations at each step, and each *β_i_* could be quite different. This is particularly important for sequential disease processes, because it emphasizes that the same risk factor could have a different influence at different stages of the disease, that in practice could produce different associations at different ages. A final observation is that because individual diseases are rare over a human lifetime and the incidence of diseases that are studied are similar, ranging between a few 100 to a few 1,000 cases in the UK Biobank cohort, the hazard of the observed diseases must be a similar order of magnitude. This requires (*t*/*L*)^*m*^ to be similar for all diseases where *t* ∼ 60, and leads to *m* and *L* being strongly correlated, with *m*log (*L*) being similar for the diseases studied here.

## Materials and methods

### Data sources

The UK Biobank dataset (27) was used, that involved over 500,000 men and women aged between 49 and 69 y, who were recruited during 2006 to 2010. Primary diagnoses of diseases in hospital records were considered, that were recorded with an International Classification of Diseases version 10 code (ICD-10) (29, 34). For data access, see “Data availability.”

### Diseases studied

The data set and disease definitions are described in detail elsewhere (27,35). Diseases were defined in terms of one or more four-digit ICD-10 codes, selected by two epidemiology-trained clinicians with specialities in pathology and general practice. The selected diseases were intended to be accurately diagnosed, distinct, and typically age-related causes of disease. Any coded diseases with an ambiguous underlying cause were excluded, as were diseases due to chance events or exposures such as an accident, or an infection while on holiday. To ensure that disease diagnoses had passed a threshold of severity, and were unlikely to result from undiagnosed or co-occurring disease, we focussed on diseases that were the primary diagnosis (and primary reason for hospital admission). As a compromise between avoiding confounding by prior disease, but retaining sufficient cases for a meaningful statistical study, we consider each individual’s first hospital admission for a disease in each ICD-10 chapter. As a result, there could be more than one case of disease for each individual, but the diseases will be from different ICD-10 chapters. Data were excluded if participants had a cancer other than non-melanoma skin cancer before their first assessment in the UK Biobank study (the start of the study period).

### Statistical analysis

The R software package was used for all analyses (36). Age was used as the time variable, left-truncated at entry into the study, and right-censored at either the study end or following incidence of a cancer other than non-melanoma skin cancer. Maximum likelihood estimates for parameters were obtained by numerically maximizing a left-truncated and right-censored log-likelihood for the data, using the “maxLik” package (37). When there was adjustment for potential risk factors, initial estimates were calculated using a proportional hazards model and the “survival” package (38, 39). To measure goodness of fit, Kaplan–Meier estimates to the data were compared with the parameterized models. Key to doing this correctly, is the observation that the Kaplan–Meier fit assumes *S* = 1 at the study’s start, whereas it may already be less than 1 if age is the time variable and participants join the study in middle- to old-age. The fitted survival functions were used to estimate *S*(*t*_1_) = exp (−*H*(*t*_1_)), where *H*(*t*) is the cumulative hazard function and *t*_1_ is the age at the first observed event, by firstly writing

(4) $$\begin{eqnarray*}
\begin{array}{ll}H(t) &=\int _0^{t_1} h(s) ds + \int _{t_1}^t h(s) ds \\ &=H_1 + \hat{H}(t), \end{array}
\end{eqnarray*}
$$

and noting that the Kaplan–Meier estimator approximates $\hat{S}=\exp (-\hat{H}(t))$. Then with the parameterized model for *H*(*t*), and the Kaplan–Meier estimate for $\hat{H}(t)$, we can rearrange Eq. 4 and average over the data to estimate

(5) $$\begin{eqnarray*}
H_1 \simeq \frac{1}{n} \sum _{i=1}^n \left( H(t_i) - \hat{H}(t_i) \right).
\end{eqnarray*}
$$

The Kaplan–Meier estimate can then be adjusted to account for *S* ≠ 1 at the study’s start, with $S(t)=\exp (-H_1)\exp (-\hat{H}(t))$. Without recognizing the need for this adjustment, and doing so correctly, the incidence of most diseases in UK Biobank would appear very differently, and would wrongly suggest that multistage diseases are very rare. Note that there are no additional free parameters introduced by the estimate. The parameterized fits were compared with the Kaplan–Meier estimator using standard χ^2^ tests, using the estimated variance at each point from the Kaplan–Meier estimate. To provide a stricter requirement for inclusion in the subsequent analysis, the fit’s variance was not used in the comparison. Diseases were excluded if there was a statistically significant difference after a false discovery rate (FDR) multiple-testing adjustment (FDR will exclude more diseases than a Bonferroni adjustment), or if the estimated effective number of steps *m* was <0.8.

All analyses were considered in men and women separately. For studies with adjustment, we considered common established risk factors of: diabetes (no, yes), smoking status (never, previous, or current), alcohol consumption (rarely—less than three times per month, sometimes—less than three times a week but more than 3 per month, regularly—three or more times each week), education (degree level, post-16 but below degree, to age 16 or unspecified). We considered tertiles of deprivation, and separately in men and women, tertiles of height and BMI. For women, we also adjusted for hormone replacement therapy (HRT) use ever (yes, no), and one or more children (yes, no). Baseline was taken as: no diabetes, never smoker, sometimes drink, minimum height tertile, middle BMI tertile, degree-level education, minimum deprivation tertile, and women with no children or HRT use. The age ranges in UK Biobank data were too narrow to allow stratification by birth cohorts, but as noted earlier, ratios such as *H*(100)/*H*(50) are determined by *m* alone and are expected to be less susceptible to cohort effects. Analyses were multiply adjusted. The data had less than 1% missing values, allowing a complete case analysis.

R packages used during the analysis, to manipulate data, and to create plots and tables include: maxLik(37), survFit(38,39), grr(40), data.table(41), bit64(42), pracma(43), and fmsb(44).

## Results

### Goodness of fit - consistency with multistage models of disease

The Weibull and NSM models both accurately fitted the incidence of most of the 800 diseases considered, with 485 with Weibull, 466 with NSM, and 450 diseases included in both sets (see Materials and Methods, and the Supplementary Material, for details and example plots). The causes of poor fits may reflect: insufficient data, diseases that were likely to have afflicted some of the cohort before electronic health records were recorded, or incidence curves that could not be approximated well by one of the models considered. For most diseases, despite the similarly good fit to the data by both the Weibull and NSM models, they require different numbers of steps in a multistage model of disease. This demonstrates that a good fit to the data with a multistage model, cannot on its own, be taken as indication of an underlying multistage biological process. The standard error of the effective number of steps was greater than 0.35 for 365 (417) of the fitted diseases for the Weibull (NSM) models, respectively, making it unlikely to see clear indications of integer-valued *m* for either model. A plot for the fitted values of the effective number of steps *m*, ordered by *m*, showed no visual evidence for steps at integer-values (Supplementary Material, Fig. S7). Neither was there any evidence for a systematic reduction in *m* for smokers, as you would expect if smoking reduced the number of steps needed to trigger disease (Supplementary Material, Fig. S8).

### Sporadic versus late-onset disease

In contrast with proportional hazards and logistic regression models, the parametric models allowed extrapolation to compare the risks of disease (first occurrence of disease in each ICD-10 chapter), at ages 50 and 100. Only diseases whose incidence data were sufficiently well fitted by a multistage model were considered (as discussed above). Fig. 2 shows the cumulative probability distribution function *F*(50) = 1 − *S*(50), for having had a disease by age 50, versus the relative increase in disease risk by age 100, (*F*(100) − *F*(50))/*F*(50). Whereas *F*(50) describes the risk of disease before age 50, (*F*(100) − *F*(50))/*F*(50) characterizes the rate of change in risk of disease with age. The probabilities were estimated with a Weibull model, and the NSM model gave almost identical results (see Supplementary Material, Fig. S2). Diseases with a late age of onset are near the top of the figure, where disease risk increases rapidly at older ages. These contrast with diseases near the bottom of the figure, whose risk increases comparatively slowly. Diseases with the lowest risk-at-age 50 are towards the figure’s left, and those with highest risk are towards the figure’s right. Diseases at the bottom right, have a comparatively high probability of occurrence by age 50 y (*F*(50) was larger than for most diseases), and the increased probability of disease-at-age 100 compared to age 50 is comparatively low (*F*(50)/*F*(100) is higher than for most diseases). (*F*(50)/*F*(100) is the probability of disease by age 50, given that you will have the disease by age 100.) Therefore, we distinguish between the most “sporadic” and “late-onset” diseases by the product *F*(50) × *F*(50)/*F*(100), that is largest (smallest), for the most (least) sporadic diseases. The diseases were classified into tertiles in Fig. 2, with most sporadic (orange), mid-range (green), and late-onset (red). The classification enabled a qualitative comparison between the late-onset and sporadic diseases.

Fig. 2 also shows the “sporadic” and “late-onset” diseases in terms of ICD-10 chapters. The late-onset diseases are composed primarily of: II Neoplasms, VII Diseases of the eye and adnexa, XI Diseases of the circulatory system, and X Diseases of the respiratory system. In contrast, the more sporadic diseases are mainly composed of: XI Diseases of the digestive system, XIII Diseases of the musculoskeletal system and connective tissue, XVIII Symptoms, signs and abnormal clinical and laboratory findings, not elsewhere classified. Similar results were found when men and women were considered separately (see Supplemental Material, Section D).

### Relative aging rates and modifiability of risk

The Weibull model has the form of a proportional hazards model, which makes adjustment for variables such as smoking status, easy to interpret. The Weibull model also allows the definition of a relative aging rate *e*^β*X*/*m*^ (see Eq. 2), that when multiplied by your age, gives your effective age for each disease in terms of your risk factors *X*, using the estimated coefficients *β* and exponent *m* for each disease. This provides an alternative measure to relative risk. Whereas relative risk measures your risk at a given time relative to the baseline, the relative aging rate allows your effective age to be determined in terms of your risk factors.

**Fig. 3.**
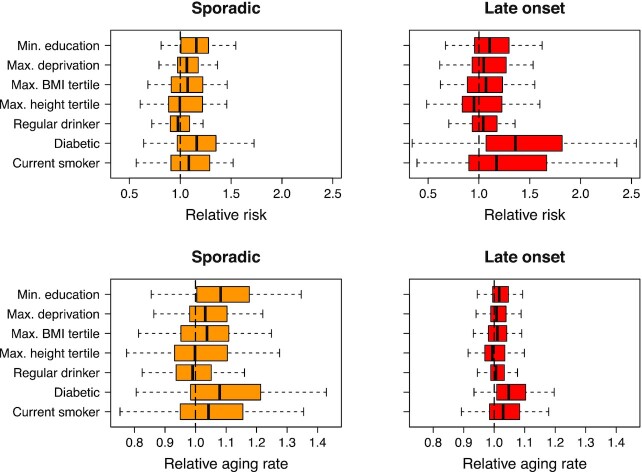
Relative risks (top), and relative aging rates (bottom), for potential risk factors associated with sporadic diseases (left), and late-onset diseases (right). Box plots show the median, the interquartile range, and whiskers at 1 × the interquartile range. Relative risks for diabetes and smoking tended to be larger for late-onset disease. Relative aging rates tended to be larger for sporadic diseases, suggesting that your effective age-at-risk is more modifiable for sporadic than late-onset diseases. After an FDR multiple-testing adjustment, a t-test identified statistically significant differences in mean values at the 0.05 level, for all associations except: height, or between regular drinking and sporadic disease.

Fig. 3 considers sporadic and late-onset diseases, and shows box plots for relative risks and relative aging rates. Comparing relative risks, the influence of diabetes and smoking were more important for strongly age-related diseases, but the results are otherwise similar. In contrast, the relative aging rates estimated for sporadic diseases were substantially larger than for diseases with a late-onset, with the relative aging rates associated with minimum education, maximum BMI, and diabetes, all being of order 1.1, and diseases with a late-onset typically less than half that. A relative aging rate of 1.1 would indicate that someone aged 50 would be at equivalent risk to someone aged 55 y with baseline risk factors. For a diabetic, in the maximum BMI tertile, and minimum education group, the relative aging rate could easily be 1.3, indicating that someone aged 50 y would be at equivalent risk to someone aged 65 without these risk factors, or someone aged 70 would be at equivalent risk to someone aged 91. In terms of disease-free years (45), if someone with baseline risk factors aged 40 was expected to have 25 disease-free years, then someone with a relative aging rate of 1.1 would on average reach (40 + 25)/1.1 ≃ 59 y before their first disease (19 disease-free years from age 40).

### Stratification by smoking status and diabetes

**Fig. 4.**
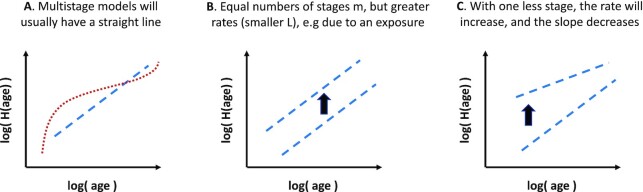
Multistage disease processes will usually have a straight line when log (*H*(*age*)) is plotted versus log (*age*) (**A**) If an exposure increases the rates of disease processes, but the number of stages are unchanged, then a plot for the exposed group will be displaced vertically upwards. (**B**) If an exposure increases the rates of one or more processes sufficiently to reduce the number of rate-limiting stages *m*, then the exposed group’s plot will be displaced vertically upwards and reduced in slope. (**C**) In a proportional hazards Weibull model with *H* = *e*^β*X*^(*t*/*L*)^*m*^, then log (*H*) = β*X* + *m*log (*t*/*L*), and risk factors *X* displace lines vertically.

**Fig. 5.**
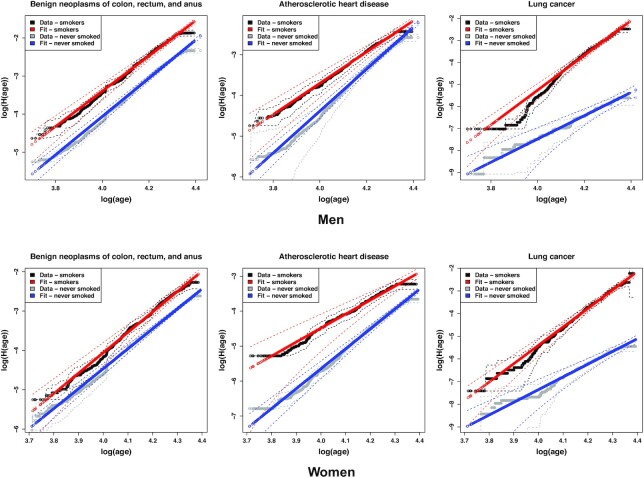
The incidence of three common smoking-related diseases in men (top) and women (bottom), stratified by smoking status, with Kaplan–Meier estimates adjusted using Eq. 5. Data for smokers differ by vertical displacements and changes in slope, that can be interpreted as changes in the rates of processes, and numbers of steps in disease (see main text). Dashed lines indicate 95% confidence intervals.

Assuming the existence of a multistage disease process, then qualitative information about the influence of an exposure or risk factor on disease can be inferred by stratifying the data by smoking status for example, and understanding how to plot and interpret the data. For the cumulative hazard function of a Weibull distribution *H*(age) that is adjusted for the survival function being less than 1 at a participant’s entry into the study, a plot of log (*H*(age)) versus log (age)) will appear as a straight line (Figs. 4A and 5, and Eq. 2). If the Weibull distribution represented a multistage model of disease, and the rates of one or more stages in the disease process were increased, then the plot would be displaced vertically upwards (Fig. 4B). If the rate of one or more stages increased sufficiently that they were no longer a rate-limiting step (Section B of Supplementary material), then the plot would be displaced vertically upwards and reduced in slope (Fig. 4C). If the rates of each stage were being decreased, then the opposite would happen, with a vertical displacement of the plot downwards and possibly associated with an increase in slope if the rates of one or more stages were decreased sufficiently. Therefore, if we stratify by strong risk factors such as smoking or diabetes status, then we can explore how these risk factors qualitatively modify disease risk under the hypothesis of an underlying multistage model (Fig. 5, and Figs. S4–S6 in the Supplementary Material). The plots were stratified by smoking status (or diabetes), but were not adjusted for any other potential risk factors.

The influence of smoking was strongly disease-dependent. Fig. 5 illustrates the differences. For benign bowel cancers (D12), the vertical displacement of plots between smokers and non-smokers is consistent with smoking increasing the rate of one or more processes that are leading to the disease, and the stratified data appear as parallel lines. For atherosclerotic heart disease (I25.1), the increased rate and reduced slope for smokers is consistent with smoking leading to an effective reduction in the number of steps needed to trigger disease, and the stratified data appear as lines that converge with age. However, for lung cancer (C34), the disease that is most strongly influenced by smoking, both the rate and slope of the curve increase, with the stratified datasets diverging with age. Reasons for this unexpected increase in slope (effective number of steps), that are specific to lung cancer, are discussed later.

Disease risks associated with diabetes could be driven by shared genetic or other risk factors, or mediated by diabetes. As an example, three common cardiovascular diseases affecting men and women were considered (Supplementary Material, Fig. S4). Atherosclerotic heart disease and atrial fibrillation are consistent with a multistep disease process in which diabetes increases the rates of disease processes, possibly sufficiently to reduce the number of rate-limiting steps needed to trigger disease. For pulmonary embolism, the rates were similar in diabetic and non-diabetic women, but there was evidence for a reduction in risk of pulmonary embolisms (I26) in the strata of diabetic men.

## Discussion

### Prevalence of multistage disease processes

Approximately two thirds of the diseases considered were consistent with a multistage model of disease, in which diseases require several distinct events, or a sequence of stages before the disease is observed. However, several authors have remarked (28) that the Weibull model in particular, would be expected to fit a wide range of models. Both the Weibull and NSM models provided good fits to the data, despite their mathematical differences and distinct biological interpretations. Therefore, based on goodness of fit to incidence data alone, it is unlikely that all the diseases have an underlying multistage aetiology.

### Stratification by smoking status or diabetes

Smoking is associated with higher risks of multiple diseases, especially cancer and cardiovascular disease. If a disease develops through a multistage process, then exposure to smoking might be expected to change the rates of one or more disease processes. As demonstrated mathematically in Section B of the Supplementary Material, if the rate of any given stage increased sufficiently, then exposure to smoking can reduce the effective number of rate-limiting steps *m* observed in the incidence data. Therefore, we might expect that if smoking was increasing the rate of one or more disease processes, then *m* should either decrease or remain unchanged. This is certainly what is seen for benign bowel cancers (D12) and bladder cancer (C67), in Fig. 5, with a vertical upward displacement of the curves indicating an increased rate of processes, and for heart disease (I25.1), that shows an increased rate and a decreased slope (decreased number of steps *m*). In these cases, the interpretation in terms of multistage processes is compelling, and may warrant further investigation.

In contrast, the influence of smoking on lung cancer incidence was initially surprising. Both the rate, and the slope (effective number of steps *m*), increase (Fig. 5). It is possible that the influence of cigarette smoke was so strong that it is entirely changing the landscape of rate-limiting steps that are needed to trigger disease. For example, if the rates of the *previously* rate-limiting steps were increased sufficiently, then there would be an entirely new set of rate-limiting processes that would determine the observed incidence data. This could produce an increased rate and an increase in *m*, and could involve several secondary smoking and age-related changes.

Importantly, lung cancer has several different types, that within ICD-10, are grouped into a single composite endpoint denoted by code C34 that was studied here. It is known that the proportions of adenocarcinoma are much higher in non-smokers than smokers, with smoking more rapidly increasing the risk of other types of lung cancer. It seems likely that different subtypes of diseases are influencing, and possibly dominating, the incidence patterns of lung cancer in smokers. This would provide one biological manifestation of a scenario that could substantially change the landscape of rate limiting processes, and the resulting incidence of disease.

If smoking is increasing the risks of different cancers to those in non-smokers, then either different processes such as different patterns of mutation in cells are occurring, or the cancers are being triggered in different cell types. A recent study of somatic mutations in the bronchial epithelium found that cells with high rates of mutations in previous smokers, are replenished over time with near-normal cells, and hypothesized that there is an ongoing replacement of progenitor cells from a pool of quiescent stem cells (46). However, smoking exposure would be greatest in the progenitor cells. If cancer initiation were solely in progenitor cells, then it would involve a competing-risk process between the cell acquiring sufficient mutations to form a cancer, and the cell’s death. Cancer risk in smokers would then be expected to saturate with age at a level where cells are being regularly replaced, with a similar statistical burden of mutations among cells. However, if smoking slowed the rate of cell turnover and death, then there would be a longer time period where cells are exposed to the carcinogenic effects of smoking. Alternately, if smoking were increasing the rate of cell turnover and death, then the increased rate of cell replication would also increase the rate of mutations occurring. Both possibilities are consistent with the suggestion that smoking may modify the rate constants for both an early and a late-stage in a multistage model of lung cancer, but not all of them (3).

The differences in incidence rates between diabetics and non-diabetics were generally consistent with the hypothesis that diabetes increases the rates of disease processes (Fig. S4 in the Supplementary Material), and this can be sufficient to reduce the effective number of steps prior to disease. An alternate explanation is that one or more genetic risk factors for diabetes are acting to reduce the effective number of steps needed to trigger disease. An interesting anomaly is pulmonary embolism in men, for which the incidence rate appears to be substantially reduced in diabetics. In contrast, for women there were no differences between those survival curves. There is no adjustment for other factors in the plots, just stratification by diabetes status at entry to the study. Nonetheless, the differences warrant further investigation.

### Comparison with Kaplan–Meier and proportional-hazard methods

Differences in incidence data can be identified by comparing conventional Kaplan–Meier survival curves, but survival curves would not appear as straight lines, and the differences would not have any particular interpretation. Equivalent plots to Fig. 5 require adjustment for the survival function being less than 1 at a participant’s entry into the study, for example, using a Weibull model for the adjustment. Differences between stratified data can subsequently be interpreted in terms of changes to the rates of processes or the number of steps, in an assumed multistage model of disease. Irrespective of whether the data reflect an underlying mechanistic process with distinct biological steps, it can be helpful to identify when the data can be modeled with several rate-limiting steps, providing a conceptual (hypothetical) model for disease onset that can be tested and explored.

Proportional hazards models are often used to estimate associations with risk factors. The proportional hazards assumption allows adjustments that correspond to vertical displacements of the curves in Fig. 5. Therefore, if adjusting for smoking with a proportional hazards model and the data considered here, Fig. 5 suggests that the model could accurately describe benign bowel cancers (D12), and approximate atherosclerotic heart disease (I25.1) or acute myocardial infarctions (I21), but it would be a poor approximation for lung cancer (C34). Whereas these differences are clear in Fig. 5, they may not be clear from a statistical test of the proportional hazards assumption.

### Can sporadic disease be avoided?

The risk of late-onset diseases increases rapidly with age, making them increasingly likely at the extreme end of observed human lifespan. In contrast, the slow increase in risk of sporadic diseases with age, suggests it might be possible to avoid them entirely. In addition, because the effective number of steps *m* is usually smaller than for the late-onset diseases, they tend to be the most modifiable by established risk factors when measured in terms of relative aging rates. For example, someone with diabetes in the lowest education and maximum BMI tertiles, has an equivalent age-at-risk of the “sporadic” diseases that is about 30% higher than someone at baseline. This would be expected to increase rates of hospitalization, irrespective of disease–disease interactions, or multimorbidity. From a statistical perspective, the results suggest that lifestyle interventions or appropriate medications might substantially reduce the incidence of a large proportion of these more sporadic diseases.

An important distinction between the diseases considered here, is whether they are curable. Whereas many of the diseases can be treated with surgery, drugs, or lifestyle interventions, diseases such as cancers have a much less certain longer-term prognosis. The sporadic diseases seem likely to have the biggest potentially avoidable impact on general health and health costs, but if you are unlucky enough to get an incurable disease then this is much more serious for the individual involved. This must be bourne in mind when deciding whether to focus extra effort on sporadic diseases to reduce hospital admissions.

### What can we learn from parametric and multistage models?

Multistage models can quantify the age-dependence of disease incidence, and provide a simple conceptual model for the progression of disease. Because two different models with very different biological interpretations, both describe the data well, any biological interpretation of the results should be treated with caution. However, the models provide a helpful picture of disease onset, that appears to have been valuable within cancer research. They also encourage us to think more carefully about modeling disease. For example, Section B of the Supplementary Material observes that a sequential progression of disease through stages could have age-dependent differences in associations, if the associations differed between e.g. an earlier and a later disease stage. Early in cancer research, a stage-dependent influence of exposures was suggested for smoking and lung cancer risk (3), but such issues are rarely considered in epidemiology. Another example in the Supplementary Material shows that if somatic mutations determine lifespan, then lifespan will be inversely proportional to the mutation rate (26).

The clearest advantage of the models used in this study, was the parametric description of disease incidence, that provided new perspectives on the age-dependent causes of hospital admission. Specifically, by being able to estimate disease risk at two very different ages, we were able to clearly demonstrate that statistically at least, some disease-risk increases rapidly with age, but others might be avoided entirely. Note that these observations were independent of the specific parameter values in the model, such as the effective number of stages *m*, only a good data fit was required.

The Weibull model’s relative aging rate also provides a more meaningful measure of risk modification than relative risk, with the former determining an effective age for each disease in terms of risk factors. A potential application when screening for disease, is to use effective age for risk stratification, by identifying an equivalent age-at-risk for groups with different risk factors. This could help to reduce over-diagnosis in low-risk groups and under-diagnosis in higher risk groups. Dedicated epidemiological studies are needed to obtain the best possible estimates, preferably involving several datasets and accounting for important genetic risk factors.

## Conclusions

Simple, biologically inspired, parametric models of disease, were found to accurately approximate the incidence rates of approximately 60% of age-related diseases in the UK Biobank dataset. Because the incidence data of most diseases were described equally well by two models with very different biological interpretations, we should be cautious about inferring that such diseases arise from a multistage process. The parametric models yielded two important new insights. The first was that the risk of some diseases increases rapidly with age, but others are more sporadic. Statistically at least, over a human lifetime we might hope to prevent the more sporadic diseases. Secondly, the Weibull model introduced the concept of relative aging rate, that provides an intuitive alternative to relative-risk for understanding how your likelihood of disease is modified by risk factors. Relative aging rates and effective ages have the potential to improve risk-stratification by identifying equivalent ages for screening groups with different risk factors. The more sporadic diseases tended to have relative aging rates that were more modifiable by established risk factors than the diseases with a late-onset in life. Overall, the findings suggest that a substantial proportion of hospital admissions for the more sporadic diseases, might be avoided by adopting a healthier lifestyle.

## Supplementary Material

pgac095_Supplemental_Files

## Data Availability

The R code that was used to produce figures from summary data, is available with the summary data at: https://osf.io/8467r. The full code for use with non-summary data will be returned with other results to UK Biobank (see www.ukbiobank.ac.uk). UK Biobank data can be accessed by application through www.ukbiobank.ac.uk. UK Biobank has approval by the Research Ethics Committee (REC) under approval number 16/NW/0274. UK Biobank obtained participant’s consent for the data to be used for health-related research.
